# Factors to Effective Telemedicine Visits During the COVID-19 Pandemic: Cohort Study

**DOI:** 10.2196/27977

**Published:** 2021-08-27

**Authors:** Kristin Nicole Gmunder, Jose W Ruiz, Dido Franceschi, Maritza M Suarez

**Affiliations:** 1 University of Miami Miller School of Medicine Miami, FL United States; 2 Department of Otolaryngology University of Miami Miller School of Medicine Miami, FL United States; 3 Department of Surgery University of Miami Miller School of Medicine Miami, FL United States; 4 Department of Medicine University of Miami Miller School of Medicine Miami, FL United States

**Keywords:** telemedicine, COVID-19, patient portals, delivery of health care, telehealth, pandemic, digital health

## Abstract

**Background:**

With COVID-19 there was a rapid and abrupt rise in telemedicine implementation often without sufficient time for providers or patients to adapt. As telemedicine visits are likely to continue to play an important role in health care, it is crucial to strive for a better understanding of how to ensure completed telemedicine visits in our health system. Awareness of these barriers to effective telemedicine visits is necessary for a proactive approach to addressing issues.

**Objective:**

The objective of this study was to identify variables that may affect telemedicine visit completion in order to determine actions that can be enacted across the entire health system to benefit all patients.

**Methods:**

Data were collected from scheduled telemedicine visits (n=362,764) at the University of Miami Health System (UHealth) between March 1, 2020 and October 31, 2020. Descriptive statistics, mixed effects logistic regression, and random forest modeling were used to identify the most important patient-agnostic predictors of telemedicine completion.

**Results:**

Using descriptive statistics, struggling telemedicine specialties, providers, and clinic locations were identified. Through mixed effects logistic regression (adjusting for clustering at the clinic site level), the most important predictors of completion included previsit phone call/SMS text message reminder status (confirmed vs not answered) (odds ratio [OR] 6.599, 95% CI 6.483-6.717), MyUHealthChart patient portal status (not activated vs activated) (OR 0.315, 95% CI 0.305-0.325), provider’s specialty (primary care vs medical specialty) (OR 1.514, 95% CI 1.472-1.558), new to the UHealth system (yes vs no) (OR 1.285, 95% CI 1.201-1.374), and new to provider (yes vs no) (OR 0.875, 95% CI 0.859-0.891). Random forest modeling results mirrored those from logistic regression.

**Conclusions:**

The highest association with a completed telemedicine visit was the previsit appointment confirmation by the patient via phone call/SMS text message. An active patient portal account was the second strongest variable associated with completion, which underscored the importance of patients having set up their portal account before the telemedicine visit. Provider’s specialty was the third strongest patient-agnostic characteristic associated with telemedicine completion rate. Telemedicine will likely continue to have an integral role in health care, and these results should be used as an important guide to improvement efforts. As a first step toward increasing completion rates, health care systems should focus on improvement of patient portal usage and use of previsit reminders. Optimization and intervention are necessary for those that are struggling with implementing telemedicine. We advise setting up a standardized workflow for staff.

## Introduction

### Background

With the rise of COVID-19 in the United States, there was a dramatic increase and widespread utilization of telemedicine—a technology that has existed for decades but represented a small fraction of care across US health systems. Telemedicine’s impetus began with National Aeronautics and Space Administration (NASA) needing to monitor the vital signs of its astronauts during manned space flights [1]. In the 1960s and 1970s, the US government funded research programs to expand telemedicine to rural areas due to a provider shortage [1]. Additional government expenditures were put toward a NASA-sponsored pilot program termed Space Technology Applied to Rural Papago Advanced Health Care (STARPAHC) that monitored Papago Indians in Arizona [1]. This demonstrated the feasibility of using the technology to provide geographically distant health care. In more recent times, Kaiser Permanente has set up, “an integrated delivery system that implemented video-visit capability for all clinicians in 2014,” allowing for use of this technology across their health system [2]. Their model demonstrated the usability of this technology to “extend established patient–physician relationships” [2]. Looking at telemedicine use beyond just the US borders, the Ontario Telemedicine Network has been one of the largest providers of telemedicine services in the world [3]. One of its aims was to increase access to underserved areas over large geographical distances, mirroring NASA’s original goals to expand access to Papago Indians. However, overall, telemedicine has been used sporadically in the United States, without major widespread adoption. With the onset of COVID-19, the health care landscape changed dramatically with patients avoiding physicians’ offices.

In order to provide quality care in an environment that allowed for social distancing and convenience, health care providers embraced the use of telemedicine. The quick scale-up of telemedicine required overcoming several barriers to acceptance and widespread usage. By expanding coverage and reimbursement, the Center for Medicare and Medicaid Services (CMS) addressed one of these issues when it announced on March 30, 2020, that it would begin covering telehealth at the same rates as in-person visits for a variety of services [4]. Other commercial insurance carriers quickly enacted similar policies; this improved reimbursement of telemedicine facilitated quick embracement of telemedicine by health care providers [4].

In addition to insurance changes, there were also Health Insurance Portability and Accountability Act (HIPAA) leniencies which allowed for more video application options to better facilitate rapid transitions to telemedicine. HIPAA enforcement was temporarily relaxed during the public health emergency (PHE), allowing providers to utilize video-calling apps such as FaceTime, Google Hangouts, and Skype, provided they were not public facing [4]. Specifically, the Office for Civil Rights (OCR) at the HHS stated they would not enforce a fine for violating HIPAA rules regarding the use of these non-public-facing audio/video applications during the COVID-19 PHE [5]. The OCR also listed vendors that claim to provide HIPAA-compliant communication including Zoom for health care [5].

Beyond the economic and HIPAA-related issues, there were further barriers to widespread implementation of telehealth by providers. Technical issues, organizational issues, and behavioral issues all played a role in reduced acceptance of telemedicine technology [6]. Many health care providers were not comfortable in acquiring and customizing this new technology workflow, nor were they sufficiently experienced in troubleshooting problems with it. Providers and support staff may not have had the time or inclination to develop the appropriate process for utilizing the technology, which typically requires organizational leadership and support. Finally, there was a challenge in terms of human behavior change. Some health care providers preferred continuing with historical procedures rather than changing their activities. While the financial and privacy-related issues were addressed, there remained these technical, organizational, and behavioral hurdles to full adoption of telemedicine by health care providers.

Regardless of the challenges in providers’ acceptance, COVID-19 brought about a rapid and unforeseen rise in telemedicine implementation for health systems. This left insufficient time for providers or patients to adapt. A recent report found that “Nearly half (43.5%) of Medicare primary care visits were provided via telehealth in April, compared with less than one percent before the PHE in February (0.1%)” [7]. A similar dramatic increase in telemedicine usage was also experienced at our institution, the University of Miami Health System (UHealth). Rapid scale-up of telehealth at UHealth occurred during the early months of COVID-19, rising to a peak of 14,852 visits per week in May, compared with an average of 17 visits per week from January until early March 2020 (Figure 2).

Regarding previous literature addressing telemedicine completion rates, some studies have examined demographics associated with completing telemedicine visits. One such study found that only 46% of scheduled patients had completed their visit, with 54% canceling or not showing. Female, non-English–speaking, older, and poorer patients in this study group had lower odds ratios (ORs) associated with telemedicine completion [8]. An additional study found that 54.4% of patients completed telemedicine visits, with older patients, Asians, non-English–speaking patients, and Medicaid-insured patients having fewer completed visits [9]. Additionally, other studies have been performed only examining no-show rates of telemedicine visits, instead of overall completion rates. While this does not directly compare with overall completion rates, no-show rates are a subset of the “incomplete” visit group. One study that had begun before COVID was able to examine no-show rates pre-COVID compared with post-COVID. They found comparable rates for telemedicine visits (9.1% pre-COVID and 8.9% post-COVID). In comparison to in-person rates for this study group, in-person no-show rates were 13.6% (in 2018) and 14.4% (in 2019) [10]. Overall, there is limited research with large-scale data sets into completed telemedicine visit rates and factors associated with them. However, we do know that telemedicine users (from pre-COVID studies) have tended to be younger, female, and live in urban areas [11]. Additionally, patients with “technology access (patients living in a neighborhood with high rates of residential internet access[)] were more likely to choose a video visit than patients whose neighborhoods had low internet access” and patients with “in-person visit barriers (patients whose clinic had a paid parking structure[)] were more likely to choose a telemedicine visit than patients whose facility had free parking.” [12].

When examining factors associated with telemedicine visit completion not necessarily related to patient demographics (ie, provider specialty or previsit reminder notifications), there is limited research investigating these strictly in relation to telemedicine. Research done pre-COVID found that visit reminders (whether automated or done by clinic staff) resulted in lower no-show rates for in-person visits [13]. Patient portal use has also been associated with improved appointment adherence and a reduction in no-show rates [14]. Provider specialties have seen differences in no-show rates throughout multiple studies conducted on different patient populations [15]. Looking at new patient appointments versus follow-up appointments, one study found a significant difference between the rate of no-shows for new patients (30%) compared with follow-up patients (21%) for in-person appointments scheduled within 30 days [16].

With all of this previous literature in mind, we hypothesized that, of course, there would be demographic drivers of differences in completion rates. However, we also conceptualized that visit reminders, patient portal use, provider specialty, and visit type (new patient vs follow-up) would likely play a role in not only no-show rates, but also overall completion rates (as no-shows would comprise part of the incomplete visit group). There is really a limited analysis of overall telemedicine completion rates in terms of characteristics that are not necessarily demographically linked. Thus, there is a need for large-scale studies that focus on aspects that could affect a wide variety of health systems that may serve different patient demographics.

### Goal of This Study

As the demand for telemedicine is likely to continue in the future, it is crucial to gain a better understanding of how to ensure completed telemedicine visits in our health system. Identifying variables that may affect telemedicine completion rates is necessary for a proactive approach to addressing various issues. While there are demographically generated disparities among patients in access to telemedicine (ie, race, ethnicity, or age that may affect access), the focus of this analysis is to highlight those changes that are actionable (ie, patient portal activation status) and can be enacted across the entire health system, regardless of the demographics of the population served.

## Methods

### Telemedicine at UHealth

UHealth main campus (located in Miami-Dade County) includes a 560-bed hospital, outpatient clinics, Sylvester Comprehensive Cancer Center, and Bascom-Palmer Eye Hospital [17]. The main campus serves a wide population from all over South Florida, but Miami-Dade County, specifically, has a population of 2,716,940 and is almost entirely classified as urban. About 69% of the population in Miami-Dade County is Hispanic and 13% are non-Hispanic Whites [18]. Additionally, in terms of satellite clinics, there are over 30 outpatient centers in Miami-Dade, Broward, Palm Beach, and Collier counties [17]. The populations in these other counties are lower (Collier County only has 384,902 people) and are more diverse in their rural–urban classification [18]. Additionally, the demographics of the satellite clinics are different in those counties outside of Miami-Dade with a lower percentage of Hispanics (23%-31%), a higher percentage of non-Hispanic Whites (35%-62%), and higher socioeconomic status [18].

**Figure 1:**
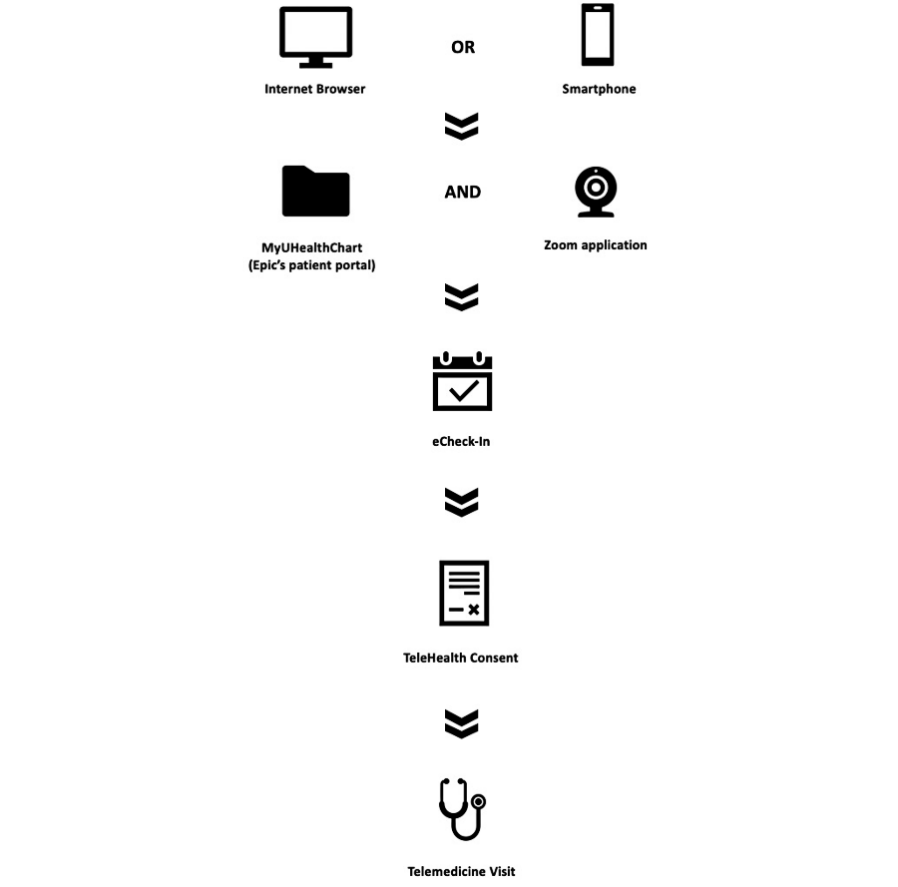
Telemedicine workflow for patients in the UHealth system. Patients must have access to an internet browser or smartphone to access MyUHealthChart and Zoom. Next, they must complete eCheck-In and TeleHealth consent prior to joining their telemedicine visit.

Within the UHealth system, Epic (Epic Systems Corporation) is used as the electronic medical record with the additional “MyUHealthChart” patient portal application. Within MyUHealthChart, patients are able to communicate (message) with their providers, view previous visit notes, examine tests results, schedule appointments, and upload Radiology images. These functions are in addition to the administrative purposes of viewing/paying bills. Specifically, to participate in a telemedicine visit at our institution, patients must go through this patient portal and perform several steps (Figure 1). Patients must first be signed up and registered for a MyUHealthChart account and log onto the patient portal via an internet browser or smartphone app. After logging into MyUHealthChart, patients must navigate to their visit and complete the eCheck-In. If it is the first visit, a consent for TeleHealth Services must be signed. Patients must download the Zoom application and click their appointment in MyUHealthChart to get to their telemedicine video visit via Zoom. In MyUHealthChart, there are videos and a guide to help patients navigate to their visit. Also, patients receive an automated appointment reminder before the visit by phone call or SMS text message based on their preferred communication method. Within MyUHealthChart, patients have access to a designated technical support number for telemedicine visit questions or troubleshooting before or during their visit. Also, the workflow is reviewed with patients on the phone with staff when scheduling the appointment and just prior to the scheduled visit. During scheduled Zoom appointments, providers are able to conduct a patient interview, but measurements (eg, blood pressure, electrocardiogram) are unable to be performed remotely. If a patient is unable to successfully access their telemedicine visit via the designated Zoom workflow within MyUHealthChart, the visit is completed via an alternative workflow such as Doximity or via a phone call (without video). Often times, this alternative workflow can occur in those that have not activated their MyUHealthChart.

In terms of implementation from the providers’ perspective, many people, processes, and technologies were organized to rapidly scale-up and expand UHealth telemedicine services. Successful telemedicine implementation resulted from multiple factors such as some providers having previously provided telemedicine services, an IT group with experience in agile workflow for quick project turnaround, and buy-in from organizational leadership. Many providers’ in-person clinics were closed by the pandemic, which allowed the associated clinic support staff to assist providers in their virtual clinics. There was not one mandated workflow, but instead there were guidelines and best practices, along with constant multimodal communication on the quickly developing policies and processes. These factors and the interest of administration and clinical workers to do what was best for the patient drove UHealth to rapidly and successfully implement a long-term strategy of telemedicine services.

### Clinical Data Collection

A clinical data request was made for all scheduled telemedicine visits (N=382,076) between January 1, 2020, and October 31, 2020. Deidentified patient-specific variables collected included age, race, ethnicity, sex, insurance, preferred language, and zip code (used to estimate income via an external data set [19]). Health system predictors collected were provider specialty, clinic location, name of provider, MyUHealthChart activation status, previsit phone/SMS text message confirmation status, new to the provider, and new to the UHealth system. All of the data were captured from the Epic system and transferred into the Clarity database, where it was pulled into exportable data sets. Data that were erroneous or had greater than 50% of the data points missing were excluded from the analysis (n=12,410). Unscheduled or “on-the-fly” telemedicine visits (n=6743) were also excluded. Deleted observations were analyzed to ensure there was no significant association (*P*>.05) between missing data and either of the completion status groups. The telemedicine visit was classified as completed if appointment status was either arrived or completed and the billing code was not null, erroneous, incomplete video, or patient left without being seen.

### Statistical Analysis

The data set was analyzed using RStudio 1.2.1335 [20] with additional packages (*furniture* [21], *lme4* [22], *ROCR* [23], and *randomForest* [24]), and visualizations were created in Tableau 2020.3.2 [25]. Data before March 1, 2020 (first officially reported COVID case in Florida) [26] were excluded from statistical tests (n=159). For descriptive statistics, continuous variables were analyzed with *t* tests and categorical variables with chi-square tests. A Bonferroni correction was utilized to adjust for multiple comparisons within descriptive statistics (α=0.05/14=.0036). Mixed effects logistic regression was used to model the completion status outcomes (α=.05) and to identify the most important system-wide hurdles to telemedicine completion. This method was used to adjust for clustering at the clinical site level, as clinical site, with 51 unique levels, was used as a random effect. The model initially included all collected patient demographic characteristics (age, race, ethnicity, sex, insurance, preferred language, estimated income, religion) that might have been possible confounders in addition to patient-agnostic variables (provider specialty, MyUHealthChart activation status, previsit phone call/SMS text message confirmation status, new to the provider, and new to the UHealth system). Using comparison of model fit statistics (Akaike information criterion and Bayesian information criterion), the model was optimized. Continuous variables were also scaled prior to modeling. Random forest was used as an additional method to examine the importance of predictors using the “importance” function to compare mean decrease Gini and mean decrease accuracy. To determine the predictive capabilities of both the logistic regression model and the random forest model, the data set was divided into a test and training set (with equal distribution of completion status between the 2 sets). Accuracy and area under the curve were assessed for both models. Data visualizations were made for individual specialties, clinics, and providers for internal use.

## Results

### UHealth Telemedicine Volume

**Figure 2:**
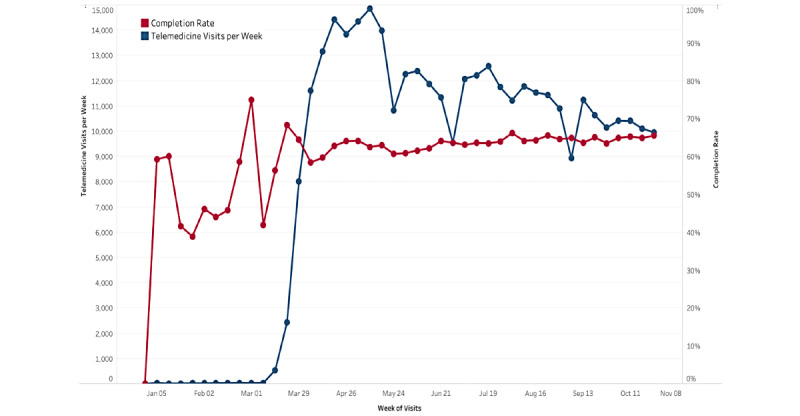
Telemedicine visits and completion rates (by week) in the UHealth system (January 1, 2020 - October 31, 2020). This figure shows the abrupt increase in telemedicine visits in the last week of March corresponding to the COVID pandemic and change in reimbursement by the CMS. CMS: Center for Medicare and Medicaid Services.

At the UHealth system, telemedicine visits began to sharply rise at the end of March 2020, at the same time completion rate leveled off from high pre-COVID fluctuations (likely high variance due to small sample size pre-COVID; Figure 2). This upward trend in telemedicine visit volume corresponded with widespread implementation and organizational support of telemedicine across the UHealth system. Interestingly, as visit counts gradually trended downward over the summer and into the fall, completion rate held steady with a minor increase from the low to mid 60% range. Over the entire period from March 1 to October 31, 2020, a total of 362,764 visits were scheduled and 230,030 visits were completed.

In terms of overall visit volume over this period, pre-COVID there were 120,403 visits (34 virtual visits) in January 2020 and 116,902 visits (46 virtual) in February 2020. Corresponding to the aforementioned rise in telemedicine visits in March 2020, 4519 of the 77,414 overall visits were virtual (5.84%). While the telemedicine visit volume continued to trend upward over the next few months, in-person visits both decreased and fluctuated substantially. In April 2020, 68.10% of overall visits were virtual (36,541/53,659 [includes both scheduled and on-the-fly]), so 17,118 were in-person visits. In May 2020, 50.50% of overall visits were virtual (36,652/72,577) with 35,925 in-person visits. In June 2020, 32.56% of visits were virtual (33,981/104,376) with 70,395 in-person visits. Over the following few months, in-person visits continued to trend slowly upward (approximately 80,000 monthly) and virtual visits accounted on average for about 25% of all visits at this time.

### Characteristics of Study Sample

The study sample mainly comprised females (217,221/362,764, 59.88%), who were White (265,451/362,764, 73.17%), Hispanic (186,268/362,764, 51.35%), having primary language as English (259,714/362,764, 71.59%), and had Commercial health insurance (209,750/362,764, 57.82%) with a mean age of 50.8 years (Table 1). Interestingly, 27.07% (98,194/362,764) of the population had Spanish selected as their preferred language.

**Table 1:** Demographic characteristics of the overall study sample and by visit completion status.

Demographics			Overall (n=362,764)		Complete (n=230,030)		Not complete (n=132,734)		*P* value
**Sex, n (%)**									<.001
	Male	145,543 (40.12)		93,038 (63.92)		52,505 (36.08)			
	Female	217,221 (59.88)		136,992 (63.07)		80,229 (36.93)			
Age (years), mean (SD)			50.8 (20.3)		50.5 (20.4)		51.3 (20.2)		<.001
**Race, n (%)**									<.001
	White	265,451 (73.17)		169,549 (63.87)		95,902 (36.13)			
	Black	45,790 (12.62)		28,464 (62.16)		17,326 (37.84)			
	Asian	6027 (1.66)		3851 (63.90)		2176 (36.10)			
	Other	3168 (0.87)		1821 (57.48)		1347 (42.52)			
	Unknown	42,328 (11.67)		26,345 (62.24)		15,983 (37.76)			
**Ethnicity, n (%)**									<.001
	Hispanic	186,268 (51.35)		115,910 (62.23)		70,358 (37.77)			
	Non-Hispanic	153,114 (42.21)		99,619 (65.06)		53,495 (34.94)			
	Unknown	23,382 (6.45)		14,501 (62.02)		8881 (37.98)			
**Language, n (%)**									<.001
	English	259,714 (71.59)		168,523 (64.89)		91,191 (35.11)			
	Spanish	98,194 (27.07)		58,732 (59.81)		39,462 (40.19)			
	Other	3756 (1.04)		2136 (56.87)		1620 (43.13)			
	Unknown	1100 (0.30)		639 (58.09)		461 (41.91)			
**Insurance, n (%)**									<.001
	Commercial	209,750 (57.82)		134,986 (64.36)		74,764 (35.64)			
	Medicare	98,737 (27.22)		62,562 (63.36)		36,175 (36.64)			
	Medicaid	43,202 (11.91)		26,383 (61.07)		16,819 (38.93)			
	Other	5366 (1.48)		3301 (61.52)		2065 (38.48)			
	Uninsured	5709 (1.57)		2798 (49.01)		2911 (50.99)			
Weighted average income (thousands), mean (SD)			97.7 (146)		100.2 (151.5)		93.6 (135.4)		<.001

Additionally, 98.67% (357,922/362,764) of visits were not new to UHealth, with only 1.33% (4842/362,764) having this visit to be their first in the UHealth system (Table 2). Concerning the MyUHealthChart (patient portal) activation status, 93.34% (338,596/362,764) of patients had activated their account, with 6.58% (23,883/362,764) not having activated it, and only 0.08% (285/362,764) having declined to have a MyUHealthChart account. Most of the visits (279,159/362,764, 76.95%) were a follow-up visit with the given provider (meaning the patient had a prior encounter within 3 years with the given provider). Clinic locations were assigned to either the main campus (217,855/362,764, 60.06%) in downtown Miami, or one of the satellite clinics (144,909/362,764, 39.95%). When grouped into 4 categories, telemedicine visits were occurring most in medical specialties (225,326/362,764, 62.11%), followed by primary care (64,164/362,764, 17.69%), surgical specialties (53,190/362,764, 14.66%), and finally in other specialties (20,084/362,764 [5.54%]; eg, optometry, audiology, exercise physiology). The patient appointment automated phone call/SMS text message reminder resulted in 38.97% (141,369/362,764) confirmed, 60.16% (218,236/362,764) not confirmed, and 0.87% (3159/362,764) answered but did not confirm (phone call only) visits.

Looking at all variables, many patient demographic characteristics had significant differences between completed and not completed telemedicine visits (Table 1). However, the focus of this analysis was to identify patient-agnostic characteristics affecting telemedicine completion rate to guide actionable changes at the UHealth system and potentially across other health systems (Table 2). For new patients to UHealth, the visit completion rate (4842/2319, 47.89%) was significantly lower than that of follow-up patients (227,711/357,922, 63.62%; *P*<.001). MyUHealthChart (patient portal) activation status also showed stark differences in completion, with activated patients completing 65.55% (221,933/338,596) of visits, while not activated or declined activation patients only completing 33.44% (7987/23,883) and 39% (110/285) of visits, respectively (*P*<.001). For patients new to a given provider, the completion rate (49,804/83,605, 59.57%) was lower than follow-up visit completion rates (180,226/279,159, 64.56%; *P*<.001). Telemedicine visits assigned to the main campus were completed slightly more often (138,994/217,855, 63.80%) compared with the satellite campuses (91,036/144,909, 62.82%; *P*<.001). In terms of completion rate based on the specialty of the provider, medical specialties had a much lower completion rate (137,195/225,326, 60.89%) than other groups, including primary care (42,388/64,164, 66.06%), surgical specialties (36,486/53,190, 68.60%), and other specialties (13,979/20,084, 69.60%; *P*<.001). Automated appointment confirmation by phone call/SMS text message was associated with a very high telemedicine completion rate (121,430/141,369, 85.90%), especially when compared with patients who answered but did not confirm or patients who did not confirm visits (63.66% [2011/3159] and 48.84% [106,589/218,236] completion rates, respectively; *P*<.001). Through more granular descriptive statistics, specific specialties, providers, and clinic locations were identified in order to provide targeted optimization.

**Table 2:** Patient-agnostic characteristics of overall sample and by telemedicine completion status.

Characteristic		Overall (n=362,764)	Complete (n=230,030)	Not complete (n=132,734)	*P* value
**New to UHealth, n (%)**					<.001
	Yes	4842 (1.33)	2319 (47.89)	2523 (52.11)	
	No	357,922 (98.67)	227,711 (63.62)	130,211 (36.38)	
**MyUHealthChart status, n (%)**					<.001
	Activated	338,596 (93.34)	221,933 (65.55)	116,663 (34.45)	
	Not activated	23,883 (6.58)	7987 (33.44)	15,896 (66.56)	
	Patient declined	285 (0.08)	110 (38.60)	175 (61.40)	
**New to provider, n (%)**					<.001
	Yes	83,605 (23.05)	49,804 (59.57)	33,801 (40.43)	
	No	279,159 (76.95)	180,226 (64.56)	98,933 (35.44)	
**Campus, n (%)**					<.001
	Main	217,855 (60.05)	138,994 (63.80)	78,861 (36.20)	
	Satellite	144,909 (39.95)	91,036 (62.82)	53,873 (37.18)	
**Specialty, n (%)**					<.001
	Primary care	64,164 (17.69)	42,388 (66.06)	21,776 (33.94)	
	Medical specialty	225,326 (62.11)	137,195 (60.89)	88,131 (39.11)	
	Surgical specialty	53,190 (14.66)	36,468 (68.56)	16,722 (31.44)	
	Other	20,084 (5.54)	13,979 (69.60)	6105 (30.40)	
**Phone reminder, n (%)**					<.001
	Confirmed	141,369 (38.97)	121,430 (85.90)	19,939 (14.10)	
	Not confirmed	218,236 (60.16)	106,589 (48.84)	111,647 (51.16)	
	Answered, not confirmed	3159 (0.9)	2011 (63.66)	1148 (36.34)	

### Modeling to Identify Important Patient-Agnostic Predictors

**Figure 3:**
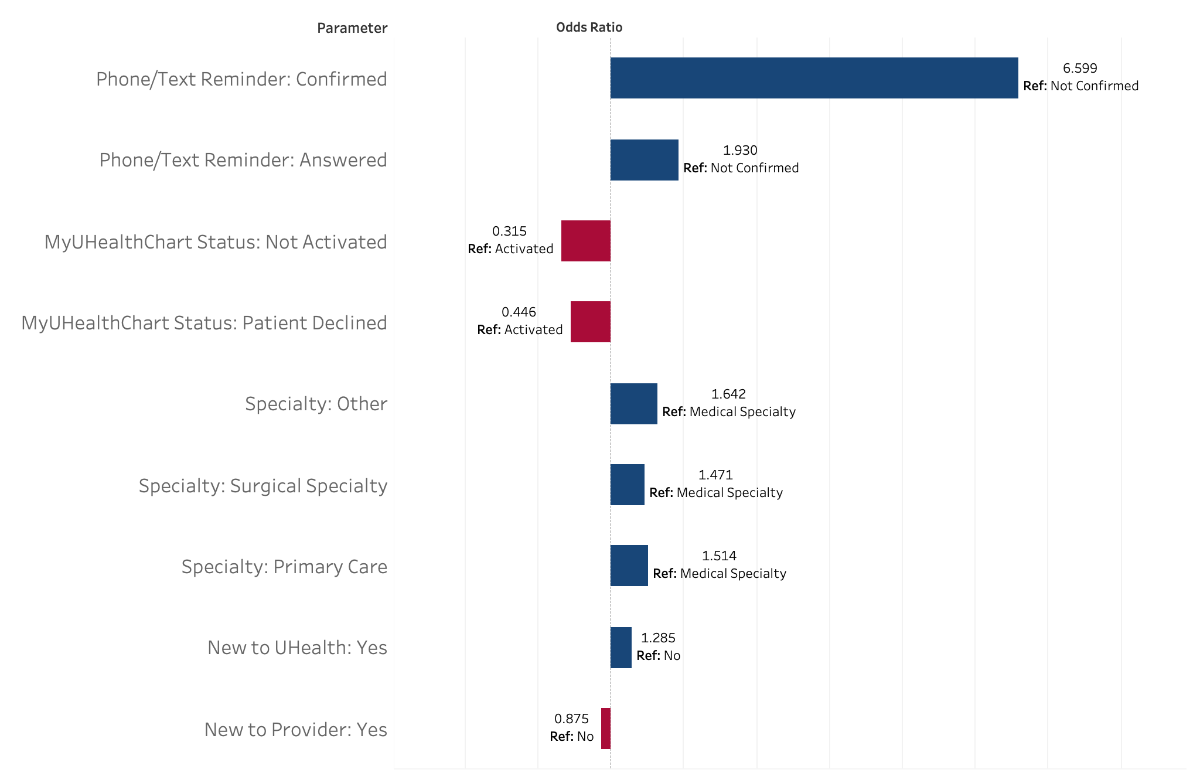
Mixed effects logistic regression model of visit completion status. These are the patient-agnostic variables (*P*<.001) that were included in the full model (which had the best fit statistics compared to reduced models). The full model also included: insurance, race, language, age, ethnicity, sex, religion, and weighted average income.

Through logistic regression (Figure 3), important patient-agnostic predictors (ie, excluding patient demographic factors) of completion included phone/SMS text message reminder status, MyUHealthChart portal status, provider’s specialty, new to the UHealth system, and new to provider. People who confirmed their appointment were 6.6 times more likely to complete their visit compared with those that did not answer the phone or SMS text message (95% CI 6.483-6.717). Even those who only answered the phone call reminder but did not confirm the visit (by pressing the prompted button) were almost twice as likely to complete their visit than those who had not answered (OR 1.930, 95% CI 1.790-2.081). Also, the MyUHealthChart portal status “not activated” had a 68.5% decrease in odds of visit completion in comparison to the activated MyUHealthChart “reference” group (*P*<.001). The MyUHealthChart status of “patient declined” was also associated with a 55.4% decreased odds of completion compared with the MyUHealthChart “reference” group (95% CI 0.344-0.577). Provider’s specialty also had a large effect on completion of telemedicine. The medical specialties group had the lowest completion and was used as the reference. Compared with medical specialties, “other” specialties had a 64.2% increase in odds; surgical specialties had a 47.1% increase in odds, and primary care had a 51.4% increase in odds of telemedicine completion compared with the reference. Being a new patient to UHealth was actually associated with a 1.285 times higher odds of visit completion compared with an established patient (*P*<.001). This may seem counterintuitive as these patients would initially be unfamiliar with UHealth’s specific telemedicine system, and descriptive statistics demonstrate that new patients fare worse than existing patients. In an unadjusted univariate analysis, the OR is less than one (0.526; *P*<.001), demonstrating that patients new to the health system have lower odds of completion. However, when used in the multivariable model and adjusting for clinical site-level clustering, the OR reverses as other potentially confounding variables are accounted for, showing the true direction of this data point. Conversely, being new to the provider (ie, not a follow-up visit) was associated with a 12.5% decrease in odds compared with being a follow-up for the provider (*P*<.001).

Random forest modeling was an additional means of verifying results from logistic regression. Using the “importance” function, the most relevant variables for predicting success in completing telemedicine visits were derived from the random forest model. These results mirrored those from the logistic model, with phone/SMS text message reminder status, MyUHealthChart status, and provider specialty being the most important in predicting telemedicine visit completion.

The predictive capabilities of both the logistic model and the random forest model were assessed. On the training data set, the logistic model had an accuracy of 69.1%, whereas on the test data set, it had an accuracy of 69.0%. This inconsequential difference in accuracy between the training and test sets indicates minimal overfitting of the model even with the large number of variables included. With regard to the random forest model, the accuracy on the training set was 71.9%, whereas on the test data set, the accuracy was 69.2%. Overall, the predictive usefulness of both of these models is quite limited given the low accuracy.

### Patients “Not Activated” in Patient Portal

The subset of “not activated” MyUHealthChart patients (n=23,883) was identified to be important because it was strongly associated with not completing a telemedicine visit, as evidenced by only 33.44% (7987/23,883) completion and 68.1% decrease in odds of visit completion compared with activated patients. This “not activated” patient portal group was investigated further and found to be demographically distinct from the rest of the population (Table 3). There was a higher percentage of males, Black patients, Hispanics, Spanish speakers, and Medicare and Medicaid patients, and they were on average older (*P*<.001).

**Table 3:** Descriptive statistics of patients with the “not activated” MyUHealthChart status.

Characteristics			Not activated (n=23,883)		Other (n=338,881)		*P* value
**Sex, n (%)**							<.001
	Male	11,196 (7.69)		134,347 (92.31)			
	Female	12,687 (5.84)		204,534 (94.16)			
Age (years), mean (SD)			51.5 (24.6)		50.8 (20.0)		<.001
**Race, n (%)**							<.001
	White	15,967 (6.02)		249,484 (93.98)			
	Black	3633 (7.93)		42,157 (92.07)			
	Asian	291 (4.83)		5736 (95.17)			
	Other	419 (13.23)		2749 (86.77)			
	Unknown	3573 (8.44)		38,755 (91.56)			
**Ethnicity, n (%)**							<.001
	Hispanic	13,128 (7.05)		173,140 (92.95)			
	Non-Hispanic	8522 (5.57)		144,592 (94.43)			
	Unknown	2233 (9.55)		21,149 (90.45)			
**Language, n (%)**							<.001
	English	13,647 (5.25)		246,067 (94.75)			
	Spanish	9694 (9.87)		88,500 (90.13)			
	Other	402 (10.70)		3354 (89.30)			
	Unknown	140 (12.73)		960 (87.27)			
**Insurance, n (%)**							<.001
	Commercial	9591 (4.57)		200,159 (95.43)			
	Medicare	7911 (8.01)		90,826 (91.99)			
	Medicaid	4699 (10.88)		38,503 (89.12)			
	Other	581 (10.83)		4785 (89.17)			
	Uninsured	1101 (19.29)		4608 (80.71)			
**New to the UHealth system, n (%)**							<.001
	Yes	2816 (58.16)		2026 (41.84)			
	No	21,067 (5.89)		336,855 (94.11)			

## Discussion

### Previsit Reminder

This analysis found that a patient who confirms his/her appointment via the automated phone or SMS text message is most strongly associated with a successful telemedicine visit completion. These results mirror what previous studies saw for in-person visits: patients who received automated reminders presented a significant difference in no-show rates compared with those that did not receive a reminder (17.3% vs 23.1%) [13]. However, it is important to note that, in this study, reminders done by clinic staff had an even lower no-show rate of 13.63% (445/3266, *P*<.01) (statistically significant at α=.05) compared with both automated reminders and no reminders. While we were unable to directly evaluate staff reminders that occurred previsit, results from automated appointment reminders are elucidating. Perhaps, these reminders allowed for confirmation with the patient prior to the visit and may have served to identify and troubleshoot technical difficulties in accessing the telemedicine visit and to provide sufficient time to ask for assistance. Also, phone or SMS text message communication may have served as a reminder of the upcoming visit that patients would have otherwise forgotten. Regardless, phone/SMS text message confirmation status is an independent critical factor to predict a completed telemedicine visit.

### Patient Portal “Activated”

The second most important variable to predict a completed telemedicine visit was having an active account for the MyUHealthChart patient portal. This underscores the importance of patients having previously activated their MyUHealthChart account prior to the visit. It is important to note that the patient portal is available in both English and Spanish. UHealth has also created multilingual telemedicine instructional videos and reference guides to best serve our diverse patient population. However, there may be underlying disparities (beyond the already addressed language barrier) to patient portal activation among certain subsets of our patient population. The “not activated” subset of patients included more Black and Hispanic patients in comparison to the rest of the sample. This mirrors results found in a study on patient portal use among older adults, which found a significant decrease in use of the patient portal among Black and Hispanic patients, in comparison to non-Hispanic White patients [27]. In addition to issues patients may face within MyUHealthChart and the Zoom workflow, there are numerous other issues which may occur. For example, patients may have internet performance issues, out-of-date Zoom applications, popup blockers, slow processors, or microphone/camera/speaker problems. A technical support line is available to patients; however, this may require additional time, patience, and technical abilities from patients.

### Provider Specialty

Provider’s specialty also played a role in completion status, with the medical specialties group, including cardiology, gastroenterology, and pulmonology, having the lowest completion rates. The highest completion rates came from other specialties, surgical specialties, and then primary care. There may be specialty-specific considerations for telemedicine which could affect completion status. “Technical and medical requirements for telemedicine differ across medical specialties;” [6]; therefore, specialties may need a custom-designed workflow to be successful, such as hybrid visits, which include on-site testing and then telemedicine evaluation. There may also be other specialty-specific barriers such as willingness to change, leadership emphasis on telemedicine, or telemedicine support allocation. As a result of urgent and rapid implementation, specialty-specific implementation and optimization were limited. This illustrates the need to reevaluate outcomes after implementation to identify opportunities for improvement across a health system.

### New to UHealth/New to Provider

Notably, new patients to the UHealth system were more likely to complete visits, which is opposite of the results seen in descriptive statistics, as additional confounders are controlled for via a multivariable model. Possibly, new patients had more time interacting with UHealth employees when scheduling their initial visit and therefore more assistance getting properly set up from a technical perspective. Concurrently, existing patients might receive relatively less previsit attention as it could be falsely assumed they had navigated the UHealth telemedicine system previously. Also, patients themselves might overestimate their familiarity with a telemedicine workflow, as they previously had an in-person visit. More research is needed to specifically examine patients new to a health system, as much of the literature focuses on new patients to providers.

New patients to a provider were less likely to complete visits compared with patients that had already established care with this provider, which is similar to results from previous studies on in-person no-show rates. One study found that, “New patients [to an academic otolaryngology department] had the highest rate of no-show [in-person] appointments” compared with other visit types (follow-up, procedure, postoperative) [28]. An additional study also found that there was a higher incidence of no-show rates (for in-person visits) among those that were new patients to a clinic (30.5%) compared with established patients (18.3%) (with *P*<.0001) [29]. Perhaps, these findings in relation to telemedicine visits could be due to the existing provider–patient relationship, which may be associated with this increase in follow-up visit completion. Established patients may be more likely to remember they have a visit and feel more accountable for attending their visit compared with new patients. Additionally, new patients may be more reluctant to seek care for a new medical issue during this PHE, which may lead to additional testing and exposure. A new patient to a provider might feel their condition requires an in-person visit and may avoid having a telemedicine visit.

### Limitations

While this analysis reveals many insights from telemedicine implementation across our health system, there are some limitations to this study and data set. Patients who canceled or did not schedule a telemedicine visit are not accounted for in this study, as we only examined those who were willing to participate in and had scheduled a telemedicine visit. As far as phone/SMS text message confirmation status is concerned, there are patients that had opted out of receiving notifications and certain visit types or specialties that had opted out of sending notifications. Therefore, there is a level to this variable that is not represented in the data which could affect results. Also, because we were provided a deidentified data set, we lacked the ability to identify repeat visits and use this information to understand how repeat visits by the same patient affect completion rate. Some providers have noted that patients who were previously unsuccessful with video telemedicine visits (having needed to convert them to telephone visits) tend to continue having difficulty with subsequent videos visits. Also, this includes data from an academic health system and does not compare with other health systems. Finally, we lacked additional variables that could serve as a better predictor of completion status and could improve accuracy of the models. Anecdotally, having a registered nurse or medical assistant help patients in navigating the telemedicine workflow was most critical to success, as there can be notable time and effort required to assist patients. Further research is needed to identify additional variables that could be used for better prediction and also take into account repeat patients.

### Future Research

Another area that was not examined was the views of providers and administrators on this technology, given that we were collecting mainly variables from the patients’ perspective. Interestingly, Tanriverdi and Iacono [6] define 4 barriers to health care providers’ acceptance of telemedicine. The technical barrier can be addressed by providing support for acquiring, developing, and customizing technology, as well as solving technical problems. The economic barrier requires the administration to develop business models that demonstrate the generation of revenue and provide a cost justification for the expense of telemedicine. This barrier also requires telemedicine reimbursement through insurance. From an organizational perspective, efforts must be exerted to create useful workflows and to provide organizational support for regular usage of the technology. Finally, from a behavioral standpoint, to be successful, telemedicine requires champions who are skilled in change management.

Additionally, as Tanriverdi and Iacono [6] state, “Experiential learning to lower the four knowledge barriers and ratification of knowledge claims through scientific and pragmatic criteria were most effective in constructing the ‘working’ of a telemedicine application.” [6]. It was expected that telemedicine visit completion rates would improve naturally over time with added provider/patient experience with this new technology. However, our analysis showed only minor improvements, indicating opportunities to progress. Likely, these completion rates could be increased by system-wide optimization compounded with reducing demographic disparities. The completion rates experienced across all disciplines may be attributable to the barriers cited by Tanriverdi and Iacono [6]. In particular, certain specialties experienced a lower completion rate potentially stemming from a lack of tailored workflows for their discipline (organizational barrier). Concerning the economic barrier, allocation of trained staff to guide patients before a telemedicine visit requires institutional finances. There are multiple barriers that can be addressed at the health system level to improve effective telemedicine overall, but more data and future studies are needed.

### Conclusions

Telemedicine will continue to be a part of delivering health care in the future, which makes it extremely important to use these results and other analyses as a guide to continued improvement. Given the current findings, an emphasis on patient portal activation and patient confirmation of appointment are high-yield changes to increasing completion rates. This ensures that not only are patients reminded of their upcoming visit, but also given sufficient time to set up the required technology. We recommend implementing a standardized telemedicine checklist for patients and staff to improve workflow. In addition, patients new to a health system may be receiving more focused previsit attention in order to better onboard them. This could possibly lead to a relative neglect of existing patients within the health system that may not be familiar with telemedicine visit procedures which differ greatly from in-person visits. All patients new to telemedicine should receive effective guidance regardless of their previous usage of the particular health care system. Attention should be paid to those specialties, providers, and locations with lower completion rates compared with others. As telemedicine was implemented on a large scale across entire health systems, certain workflows or features may not be transferrable to particular providers. These users should receive greater technology acclimation intervention, as well as be consulted regarding telemedicine workflow changes that would be appropriate for them. While telemedicine should be tailored, there also needs to be a standardized workflow for clinic staff to guide patients through the system. With these changes, telemedicine completion rates can be improved on a wider-scale, paving the way for additional technology innovation in medicine for future years to come.

## Abbreviations

CMS: Center for Medicare and Medicaid Services
HIPAA: Health Insurance Portability and Accountability Act
NASA: National Aeronautics and Space Administration
OCR: Office for Civil Rights
PHE: public health emergency
STARPAHC: Space Technology Applied to Rural Papago Advanced Health Care
UHealth: University of Miami Health System
